# Bacteriologic Studies in Acute Rheumatic Fever

**Published:** 1917-07

**Authors:** 


**Bacteriologic Studies in Acute Rheumatic Fever.** H. F. Swift and R. A. Kinsella, Arch. of Int. Med., page 382, Vol. 19, made 85 blood cultures in 58 typic cases, before the administration of salicylates. Joint cultures were all negative. In 6 cases, streptococci of the viridans group were obtained, but there seemed to be no specific organism and the patient's blood did not show antibodies against the bacteria isolated.

